# Study of the Ubiquitous Hog Farm System Using Wireless Sensor Networks for Environmental Monitoring and Facilities Control

**DOI:** 10.3390/s101210752

**Published:** 2010-12-02

**Authors:** Jeonghwan Hwang, Hyun Yoe

**Affiliations:** School of Information and Communication Engineering, Sunchon National University, Maegok-dong, Suncheon-si, Jeollanam-do, Korea; E-Mail: jhwang@sunchon.ac.kr

**Keywords:** WSN, ubiquitous, agriculture, pig, hog farm

## Abstract

Many hog farmers are now suffering from high pig mortality rates due to various wasting diseases and increased breeding costs, *etc.* It is therefore necessary for hog farms to implement systematic and scientific pig production technology to increase productivity and produce high quality pork in order to solve these problems. In this study, we describe such a technology by suggesting a ubiquitous hog farm system which applies WSN (Wireless Sensor Network) technology to the pig industry. We suggest that a WSN and CCTV (Closed-circuit television) should be installed on hog farms to collect environmental and image information which shall then help producers not only in monitoring the hog farm via the Web from outside the farm, but also facilitate the control of hog farm facilities in remote locations. In addition, facilities can be automatically controlled based on breeding environment parameters which are already set up and a SMS notice service to notify of deviations shall provide users with convenience. Hog farmers may increase production and improve pork quality through this ubiquitous hog farm system and prepare a database with information collected from environmental factors and the hog farm control devices, which is expected to provide information needed to design and implement suitable control strategies for hog farm operation.

## 1. Introduction

Recently the Korean pig industry has been forced to compete directly with those in developed countries due increased feed prices and FTA treaties, *etc.* [1], and further is suffering from high pig mortality rates caused by various wasting diseases and high production costs due to price increases for feed, other materials and energy [2]. In order to solve these problems, it is necessary for hog farmers to establish an optimal pig breeding environment through application of systematic and scientific pig breeding technology and to increase production of high quality pork by decreasing mortality rates and production costs.

In this study, we would like to propose a ubiquitous hog farm system applying RFID/WSN (Radio Frequency IDentification/Wireless Sensor Networks) technology to the pig industry in order to implement such a systematic and scientific pig breeding technology.

WSN is a technology whereby sensor nodes capable of computing and communication are deployed in various application environments so that they can form an independent network, then physical information collected by wireless from the network can be utilized for monitoring and controlling, *etc.* [3,4]. This WSN technology contributes to realizing high productivity, safety and high human quality of life through its applications in various industries such as distribution, logistics, construction, transportation, military defense and medical services, *etc.* [5,6].

Nowadays RFID/WSN technology is applied to various agricultural fields such as greenhouses and livestock to achieve high productivity and transparency of distribution routes from the cultivation environment to production management and distribution logistics, *i.e.*, a total monitoring system [7–9]. Especially in the livestock industry, RFID/WSN technology is being used for managing each animal’s characteristics, livestock shed environment and for tracking breeding history [10–12].

Mayer *et al.* created a wireless sensor network platform for animal health and behavior monitoring. A steer was equipped with both internal and external sensors, using matchbox sized motes placed inside standard drug release capsules. The nodes monitored the intra-rumenal activity of the steer and communicated wirelessly with each other [13].

Ipema *et al.* described the results of an experiment in which a temperature sensor built into a bolus was placed in the rumen of a cow. The main objective was to demonstrate that capsule-based wireless technology could work in cattle. The mote in the rumen transmitted data to the mote attached to the front leg of the cow; from there the signal was transmitted to the base station [14].

Evaluation of animal welfare can also be accomplished by wireless monitoring and enables the producer to make the right decisions based on real-time management. Nadimi *et al.* addressed and solved the problem of on-line monitoring of cows in an extended area, using ZigBee based wireless sensor networks. A study of wireless sensor networks applied to the monitoring of animal behaviour in the field is described. The problem of online monitoring of cows’ presence and pasture time in an extended area covered by a strip of new grass using wireless sensor networks has been addressed [15,16].

Monitoring and control of the quality of indoor environment is very important for animal health and welfare and directly impacts productivity and quality. Ventilation in the stables must be managed in order to avoid long-term over-critical exposure of the animals to ammonia, causing stress, pour health and reduced productivity. Cai *et al.* presented a wireless, remote query ammonia sensor that can track both low and high concentrations of ammonia [17].

At the same time, ventilation and heating must be minimized in order to save energy while keeping temperatures at an adequate level. Cugnasca *et al.* evaluated the capability and usefulness of a WSN applied to monitoring environmental variables in an animal housing facility. The nodes were moved through the facility to determine different profiles of temperature, humidity and luminosity [18]. Darr and Zhao developed a wireless data acquisition system for monitoring temperature variations in swine barns [19].

In this paper, the proposed ubiquitous hog farm system is composed of the physical layer to collect information of the hog farm and to control the environment, and the application layer which supports communication between the physical layer and the application layer, and lastly the middle layer which utilizes information of the hog farm as a database and provides monitoring and control services to keep the pig breeding environment in an optimal status.

Pig breeding environment information and external environmental information are collected through a wireless sensor network formed with sensors installed in the hog farm, and visual information about the hog farm is collected and monitored through CCTV. All this environmental and visual information about the hog farm shall help a user monitoring and controlling the hog farm facility from outside the hog farm via the middle layer.

In addition, hog farm facilities could be automatically controlled based on some breeding environment value which is already set up and SMS notice service shall be provided to users when dangerous situation occur, while RFID technology enables users to collect each pig’s identification and individual information for their efficient history management.

This study shall analyze the system requirements for monitoring and management of the hog farm in Section 2, and explains the structure and providing service process of proposed ubiquitous hog farm system in Section 3. Section 4 shall explain the results realized by the proposed system and lastly, Section 5 shall finish the study with the conclusions.

## 2. Requirements Analysis of a System for Hog Farm Environmental Monitoring and Management

It is necessary to analyze the environmental features of the hog farm and each individual animal’s behavioral characteristics in a specific environment in order to develop the ubiquitous hog farm system as proposed in the study. This chapter shall list some items which an ubiquitous hog farm system must have by examining relations between each environmental aspect and the individuals’ behavioral characteristics.

### 2.1. A Systemic Management Considering Environmental Factors

The hog farm must eliminate or mitigate environmental factors which may cause diseases through systematic management practices considering various environmental factors such as temperature, humidity and the presence of various harmful gases in order to prevent diseases and maintain an optimal breeding environment [20–22].

Next, requirements for control devices installed inside the hog farm in specific environment according to environmental factors shall be listed. For air ventilation devices, the user should maintain environmental conditions suitable for individual animal’s growth and development by reflecting environmental factors such as set temperature, temperature deviations, minimum and minimum air ventilation amounts, *etc.*, which are regularly monitored to control devices.

#### 2.1.1. Temperature Setting

As pigs grow, the temperature in the hog farm should be set at a level the pigs find comfortable, however erroneous temperature information could be provided depending on the locations of sensors, therefore a method to compare the temperature values obtained from the sensor to the ones obtained from the thermometer(s) placed in the hog farm shall be necessary.

#### 2.1.2. Air Ventilation Quantity Setting

Setting minimum air ventilation quantity is important in winter or in-between seasons, and particularly during the night, users should set minimum air ventilation quantity by observing pigs’ sleeping status according to temperature variations so they should lower minimum air ventilation quantities or supplement with heat or insulation when the internal temperature falls below some level during the minimum air ventilation phase in the hog farm. However, pigs’ sensory temperatures may fall according to the maximum air ventilation quantity in summer, but it’s not so important in winter or in-between seasons.

#### 2.1.3. Temperature Variations

The difference between maximum air ventilation quantity and minimum air ventilation quantity shall be divided by temperature deviation to determine the fan speed per temperature increase by 1 °C.

In winter and in-between seasons, the environmental variation should be minimized by setting the temperature deviation to more than 4 °C while they have to discharge the warm air to the outside by accelerating fan speed immediately when the temperature goes up by setting the temperature deviation at 2 °C in summer.

### 2.2. Environment Management Based on Individual Animals

All environmental management should control and manage internal environmental factors based on pigs, and for this a system which shall provide the hog farm with an optimal environment by controlling temperature setup, air ventilation quantity, height of heating lamps, *etc.* through careful observation of individual animals’ status shall be necessary.

Hog farms with high mortality rates have something in common in that operators don’t have a sense of standards for environment management but instead they control the environment only by the absolute temperature. However, the absolute temperature is not so important for the environment inside the hog farm but the sensory temperature of the pigs is important, so a complex and systemic change of thinking is required because a sensory temperature varies according to absolute temperature, floor material, humidity, air flow speed and radiation from heating lamps, *etc.*

#### 2.2.1. Creating a Standard Index of Specification Management of the Hog Farm

An environmental index in the hog farm and an infrastructure for scientific hog farming specification management should be prepared by establishing a standard index of the specification management through behavioral analysis of the pigs (frequency and quantity of the feed and drinking water) according to environmental changes such as season, time, days of age, *etc.*

#### 2.2.2. Increasing Efficiency of the Hog Farm Management

They should be capable of addressing any hog farm calamities immediately by monitoring the hog farm via CCTV and the ventilation air by remote control through a SMS service for noticing early signs of disorder inside the hog farm.

#### 2.2.3. Scientific Specification Management

Specification management for disease forecasting activity and selective separation treatment to save feed costs and decrease mortality rates shall be necessary by preventing feed loss caused by hogging down by providing proper feed quantities considering feed per period, drinking water, weight, *etc.*, and identifying pigs which have low intake amounts and are underweight.

## 3. Proposed Ubiquitous Hog Farm System Structure and Service Process

**Figure 1.**
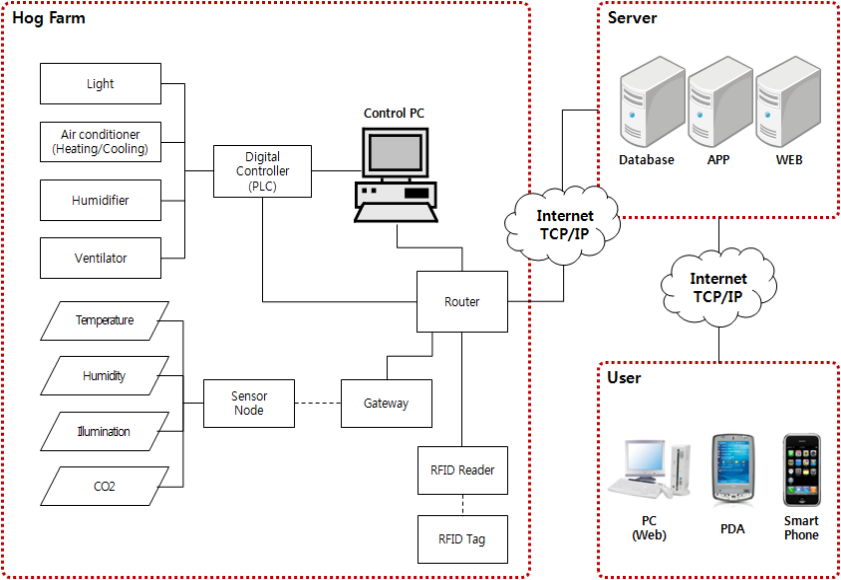
Hardware scheme of data acquisition and facilities control.

The proposed ubiquitous hog farm system shall collect and monitor the breeding environment information such as illuminance level, humidity, CO_2_ *etc.* and external environment information such as theft or fire *etc.* through WSN of sensors installed in the hog farm, and in addition collects and monitors image information of the hog farm through CCTV. The collected environment information and image information shall help monitoring the hog farm and controlling its facility outside the hog farm via management server. Further, it collects and manages identification and individual information of pigs using RFID, through which an efficient history management is possible (Figure 1).

### 3.1. System Architecture

**Figure 2.**
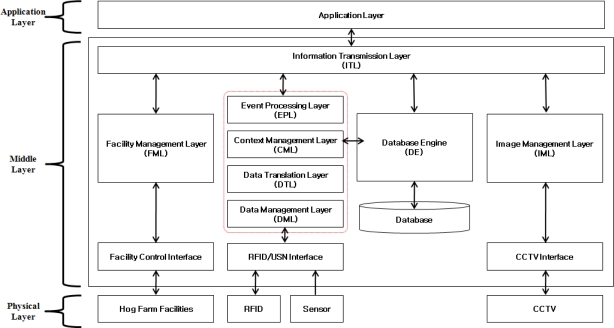
Ubiquitous Hog Farm System Architecture.

The proposed ubiquitous hog farm system shall be classified to three stages following Figure 2, and each class is composed of the physical layer which consists of environmental sensors, RFID, CCTV and other control facilities of the hog farm, and the application layer which consists of interfaces that support monitoring of the hog farm and the controlling service of the pig breeding environment, and finally of the middle layer which supports the communication between the physical layer and the application layer, and stores the information collected from the hog farm in a database, provides monitoring and controlling services while maintaining the optimal status of the pig breeding environment.

#### 3.1.3. Physical Layer

The physical layer consists of the environmental sensors which collect environmental information about the hog farm, RFID which stores the identification and history information of the pigs, CCTV which collects image information about the hog farm, and the hog farm control facility which establishes and maintains an optimal breeding environment.

The environmental sensors shall be installed both inside and outside the hog farm to collect the information such as illumination level, humidity, temperature, CO_2_ levels, *etc.* and external environment information such as theft and fire, *etc.*, and each sensor node shall form an independent network which shall collect physical information obtained from each sensor node and measures any environmental variation.

For RFID, a tag will be attached on each pig to maintain information about individuals’ identification and growth, which shall be read via a RFID reader. RFID shall collect all the foregoing information or revise changed information.

CCTV shall be installed both inside and outside the hog farm; the internal system will collect image information about the hog farm and individual pigs and the external one shall be installed to prevent dangers from events such as theft and fire.

The hog farm control facility refers to devices which control the environmental factors such as illumination levels, temperature, humidity and CO_2_ that may affect a pig’s growth and development, and consists of lightening apparatus, humidifiers, air conditioners and air ventilators, *etc.*, and each hog farm control facility shall be controlled through a digital controller.

#### 3.1.2. Middle Layer

The middle layer has features to collect data occurring in the physical layer such as sensors installed for monitoring the hog farm environment, RFID for managing information of each pig’s identification and individual history, and to lower the load on the application programs by filtering real time information and provide data requested by the application programs.

In order to meet all these requirements, the middle layer has functions to refine, filter and convert the collected information and support functions to recognize the situation and process and control the respective situation information when an event occurred.

DML (Data Management Layer) plays the role of filtering data and delivering valid messages to its higher layer in order to lower the load of the great amount of collected information obtained from RFID, sensors and load cells, *etc.*, on application programs.

DTL (Data Translation Layer) converts messages filtered from management layer to a data format suitable for use in application programs.

CML (Context Management Layer) infers the status of a relevant event and the patterns of its surrounding environment through the inference engine by referring information on various individuals obtained from collected data.

EPL (Event Processing Layer) forms and processes the event based on inferred information from the context management layer, and normally controls the hog farm control facility automatically or processes the emergency alarms in dangerous situations.

DE (Database Engine) stores real time data into the database and supports queries from the application layer in order to use data in a timely way, and stores or updates the standard data for automatic control of the hog farm control facility and its status notices.

FML (Facility Management Layer) converts the control signals transmitted from the application layer or EPL to a proper typed data format, and transmits it to the hog farm control facility to control, and transmits the status, operation time and control frequency of the control facility to DE to store them in the database.

IML (Image Management Layer) provides the Web with stream data of images taken from the CCTV system and classifies them according to the hog farm ID and camera number, *etc.*, to be stored in the data base.

ITL (Information Transmission Layer) plays a communication role between the middle layer and the application layer providing application programs such as the hog farm environmental monitoring service, the hog farm facility control service, pigs’ history management service, *etc.*, with data.

The database plays the role of saving in its tables the hog farm environmental data collected from sensors installed inside/outside the hog farm, image data collected from CCTV, information about identification and individuals collected from RFIDs and load cells, status, operation time and control frequency of the hog farm control facility, and base environmental values for automatic control and status notices.

#### 3.1.3. Application Layer

The application layer consists of application services which support various platforms such as laptops, the web, PDAs and smart phones, *etc.*, and provides users with the hog farm monitoring service, the hog farm facility control service, pig history management service and situation notice services, *etc.*

### 3.2. Service Process

The proposed system provides users with a hog farm monitoring service with which they can monitor the hog farm environmental and image information, a hog farm facility control service for optimal management of the pig breeding environment, the pig traceability service with which they can monitor information about a pig’s history and individual characteristics, and a situation notice service to inform about any danger to the hog farm when emergency situations occur.

#### 3.2.1. Hog Farm Monitoring Service

The hog farm monitoring service is a service which enables pork producers to monitor through a GUI important breeding environment information such as illumination levels, humidity, temperature, CO_2_, *etc.* which were collected by environmental sensors and CCTV, and external environment information such as theft and fire, and image information of the hog farm. This service can be divided into the hog farm environment monitoring system and the hog farm image monitoring service and the detailed operation of each service are as follows.

**Figure 3.**
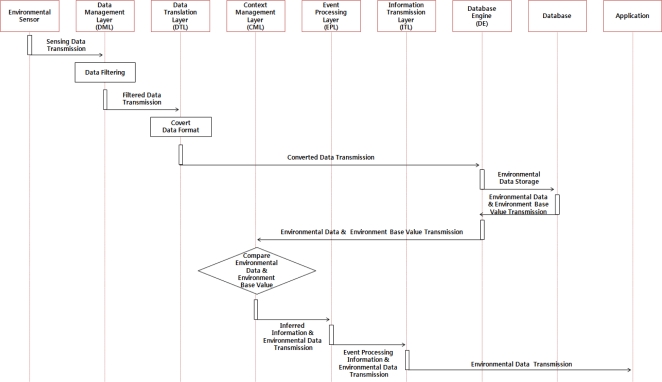
Operation of the Hog Farm Environment Monitoring Service.

When examining the detailed operation process of the hog farm environment monitoring service, environmental sensors installed at the hog farm collect the environmental data of the farm and deliver it regularly to the DML. Then the DML delivers valid messages to the DTL after filtering the collected information, and the DTL converts the filtered information to a data format suitable for use in the application program(s). The converted data shall be saved into the database through the DE and delivered to the application layer via the CML and EPL so that a user can monitor the hog farm’s environmental information. Figure 3 below illustrates the operation of the hog farm environment monitoring service.

**Figure 4.**
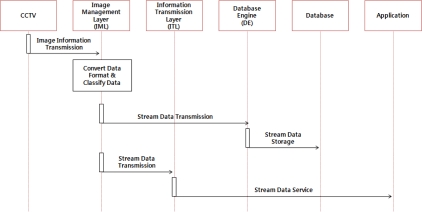
Operation Process of Hog Farm Image Monitoring Service.

In addition, the image monitoring service of the hog farm collects image information from the CCTV system installed in the hog farm and transmits it to the IML, which provides the application layer with a stream of data and stores it in the database via the DE after classifying the stream of data according to the hog farm ID and camera number. Figure 4 shows the operation process of the hog farm image monitoring service.

#### 3.2.2. Hog Farm Facility Control Service

The hog farm facility control service is a service which controls the hog farm control facility automatically in order to maintain an optimal pig breeding environment based on the collected information from the hog farm or helps users controlling the facility manually.

**Figure 5.**
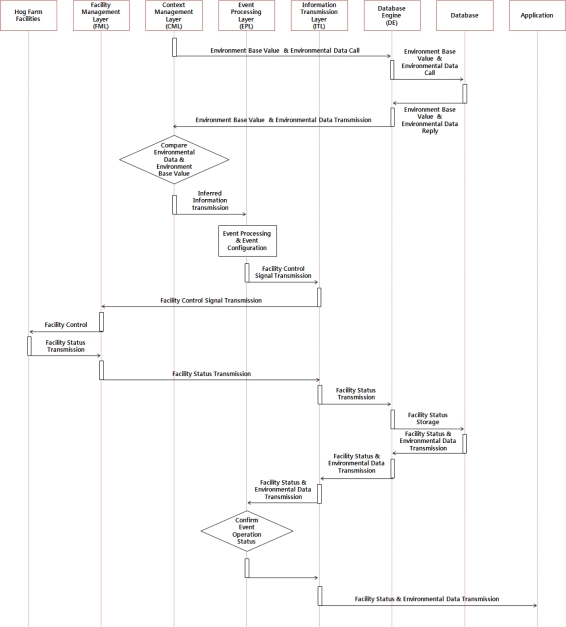
Operation Process of Hog Farm Facility Automatic Control Service.

For the automatic control service, CML compares the collected information to the base environment values stored in the database via DE and infers the situation of the relevant event and the patterns of its surrounding environment. In addition, CML confirms the operation of the hog farm’s control facility based on the information inferred from the EPL and transmits the control signals to the FML so that the hog farm’s control facility can respond appropriately to the given situation given, then informs all users via the application layer. Figure 5 shows the service operation process of the automatic control service of the hog farm facility.

**Figure 6.**
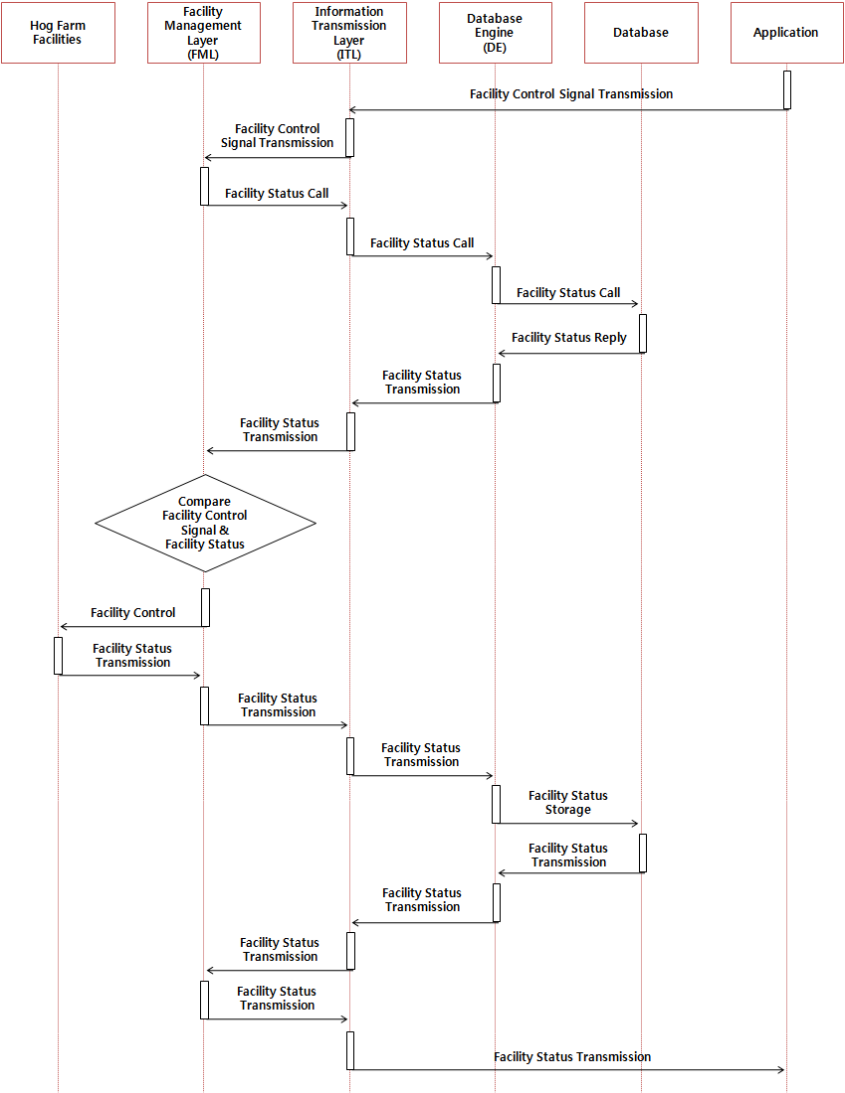
Operation of the Hog Farm Facility Manual Control Service.

The manual control service is a service which supports users when they would like to control the the hog farm facility after checking the hog farm information such as breeding environment information, external environment information, image information of the hog farm, *etc.* When users transmit a control signal to the FML via the GUI and ITL, the FML then checks the operation of the facility stored in the database via DE and converts the transmitted control signal from the application layer to the proper typed data format and transmits it to the control facility of the hog farm. Figure 6 illustrates the operation of the manual control service of the hog farm.

#### 3.2.3. Pig Traceability Service

**Figure 7.**
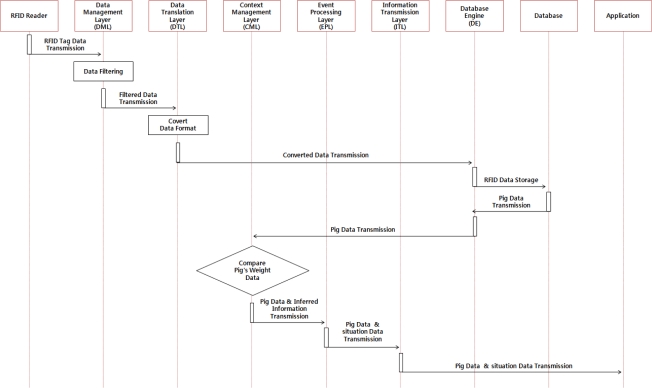
Operation Process of Pig Traceability Service.

The pig traceability service is a service that helps users control the hog farm efficiently by monitoring pigs’ history and individual information. It collects identification and individual information about the pigs through the RFIDs tag attached to the pig and the collected data shall be filtered by the DML to obtain valid messages which shall be delivered to the DTL. Valid messages shall be then converted to a suitable data format for use through the DTL and be stored in the database via the DE to be delivered to the application layer via the CML and EPL so that users can monitor them. CML can call daily information about pigs’ weights measured by load cells from the database and compare them. When the variation range exceeds a certain level, it is inferred that there is a high potential of animal disease occurrence and the inferred information shall be delivered to users via the EPL. Figure 7 illustrates the operation of the pig traceability service.

#### 3.2.4. Danger Situation Notice Service

**Figure 8.**
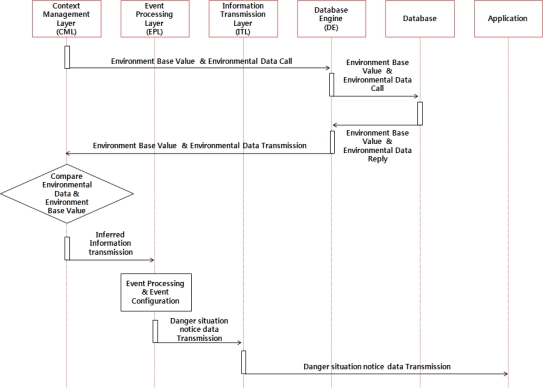
Operation process of Danger Situation Notice Service.

The dangerous situation SMS service is a service that informs users like farmers of weather changes or any situation changes so that users can take necessary actions to prevent such a dangerous situation. CML infers the situation of the relevant event and patterns of its surrounding environment after comparing collected information from the hog farm to the environment base values of the database. Then the EPL inform users of the factors of the event via SMS service based on the inferred information. Figure 8 below shows operation process of the danger situation notice service.

## 4. Implementation of the Proposed Ubiquitous Hog Farm System

### 4.1. Environmental Sensors

**Figure 9.**
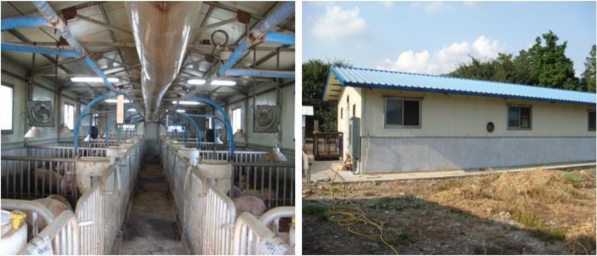
Installation sites of the Ubiquitous Hog Farm System.

Environmental sensors are installed in the hog farm as shown in Figure 9 in order to collect environmental information about the hog farm, and these sensors in general consist of WSN environmental sensors and wired sensors.

We designed and realized low power and low cost small sensor nodes in order to apply them to WSN environmental sensors operating in wide range sensor networks. The sensor node receives the sensor data from temperature, humidity sensors, *etc*. It processes the data at the MSP430 MCU [23] and transmits them to relay nodes and the gateway, using the CC2420 RF chip [24]. In order to reduce the impact of any heat the sensor receives from the node, the node and the sensor will be placed at a certain distance from each other.

MSP430 is a 16 bit RISC with 48 Kbyte program memory and 10 Kbyte RAM inside. It can process multiple sensor data at high speed. CC2420 is an RF chip supporting Zigbee. It supports the 2,400 ∼ 2,483.5 MHz frequency band. It operates in DDDS method, supports O-QPSK modulation method and 250 kbps baud rate. It enables real time wireless communication with small power consumption. Power is provided to the sensor nodes by using TEKCELL 5.6 V batteries, and the TK71750 LDO is used for supplying stable power to nodes.

As temperature and humidity sensor, the SHT71 is used, which combines both functions in an all-in-one style. The operation power is 2.4 V ∼ 5.5 V, which is relatively low, and power consumption is low as mean 28 μA. The inside of the sensor has an offset memory, 14 bit A/D converter and digital 2-wire interface, and the temperature can be measured over the range −40 to 120 °C, with an error of 0.5 °C. In addition, humidity is measured between 0 and 100% with an accuracy of 3.5%.

**Figure 10.**
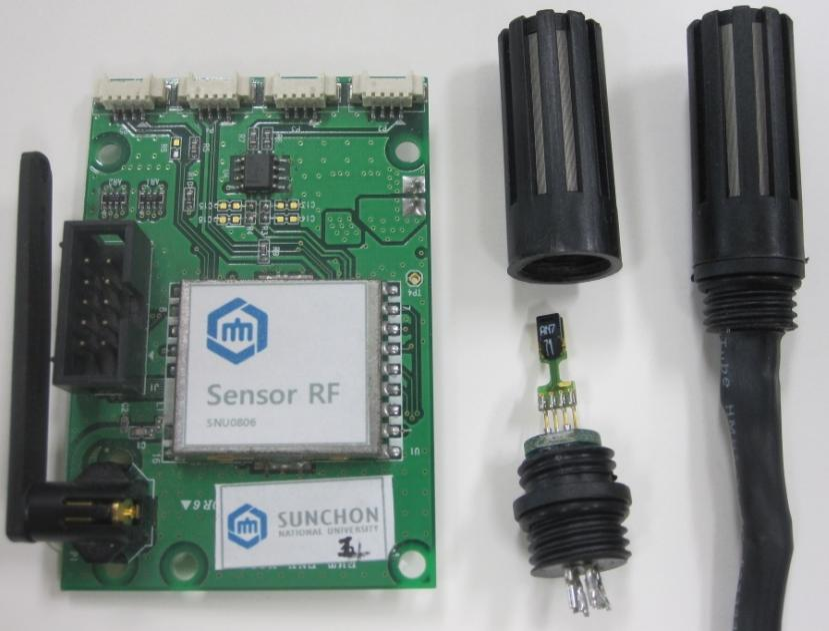
WSN Environmental Sensors.

We connected the 3.3 V operation voltage to the sensor nodes and connected the digital 2 wires to the MSP430 circuit to process temperature and humidity date from the hog farm, and these sensor nodes forms a wireless network together with the WSN sensor gates of the hog farm. The following Figure 10 and Table 1 indicate the hardware types and the specifications of the WSN environmental sensors installed at the hog farm.

**Figure 11.**
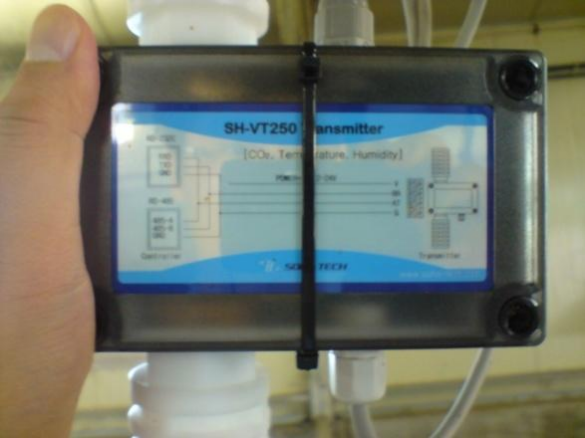
Wired Integrated Sensors.

In addition, since the environmental sensors of the hog farm may provide wrong information about the hog farm, we compare it to environmental information values obtained from wired sensors which were installed in the hog farm too. The wired sensors can measure CO_2_, temperature and humidity, *etc*., For this NDIR CO_2_ Dual Sensor type sensors are used so no calibration is necessary for long periods and RS-485 is used as the communication method. Figure 11 and Table 2 show the hardware types and specifications of the wired sensors installed at the hog farm.

### 4.2. RFID Tag and Reader

**Figure 12.**
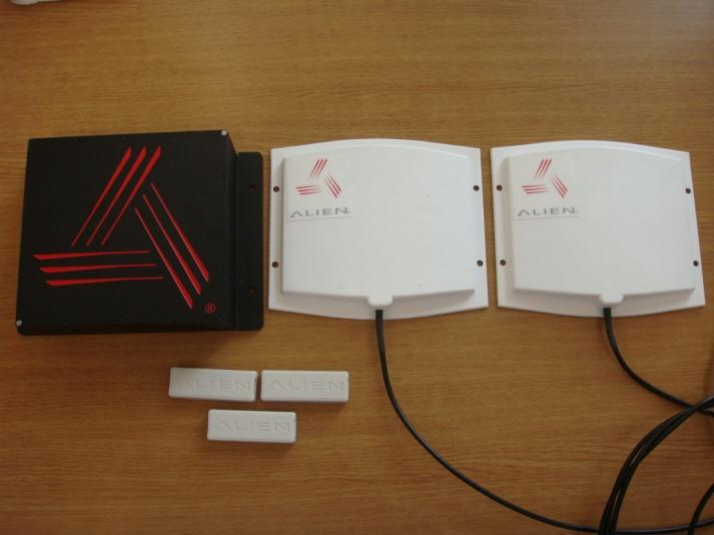
RFID Tag and Reader.

RFID consists in general of a tag which keep information about the identification, individual characteristics, and growth of the pig and an RFID reader which collects the information of the tag or revises information when it changes. Users can monitor a pig’s history through the RFID. The Alien 2850 BAP Dev Kit was used to monitor pigs’ histories and the RFID used in the proposed ubiquitous hog farm system and its specifications are shown in Figure 12 and Table 3.

### 4.3. CCTV System

**Figure 13.**
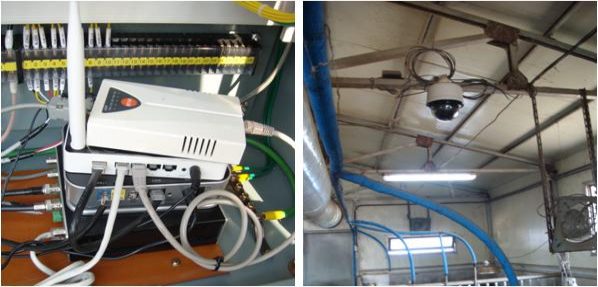
DVR and CCTV.

Surveillance cameras were installed to monitor image information inside and outside the hog farm for 24 hours (Figure 13). These cameras, with their 24 hours recording function, shall be used for finding causes when events like a theft or accident occurs or are used for checking the status of the hog farm and animals in real time. Images taken shall be transmitted to the ML and stored into the database after being classified according to the hog farm ID and camera number, *etc.*

### 4.4. Hog Farm Facility and Digital Controller

**Figure 14.**
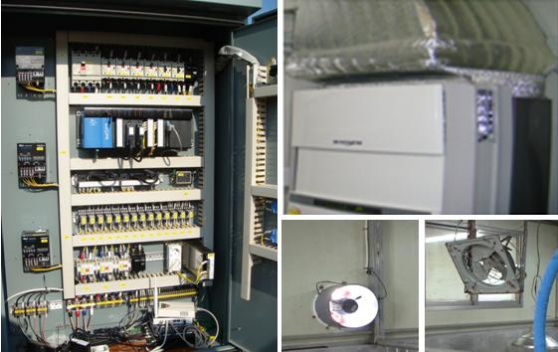
Digital Controller (PLC) and Hog Farm Facilities.

Luminosity, temperature, humidity and CO_2_ impact the growth of pigs. Figure 14 shows the environmental control devices in the hog farm, which enable the control of hog farm facilities such as lighting, humidifiers, fan heaters, air conditioners and ventilators. The internal breeding environment shall be controlled to a pleasant level for the pigs through environmental control devices and diseases affecting pigs’ growth and development can be prevented.

### 4.5. Application Program

GUI for the data manager is developed for a Web environment. Tomcat-6.0.20 is used for WAS and ‘mysql’ is used for the database. The latest released version 5.0 was used.

**Figure 15.**
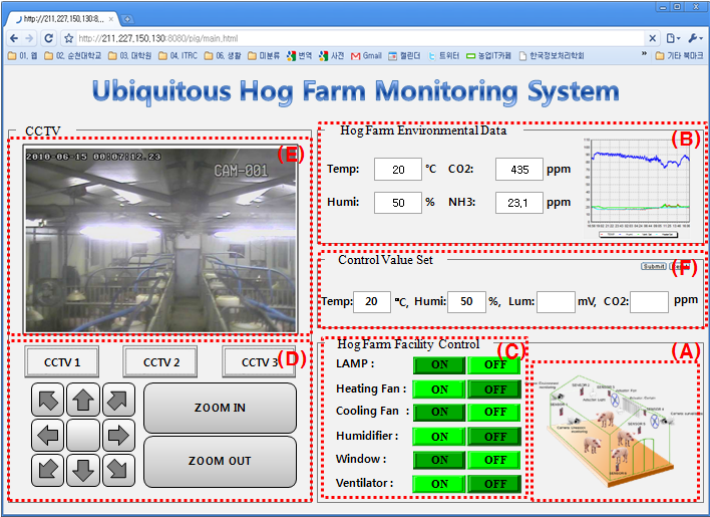
Ubiquitous Hog Farm Monitoring System GUI.

Figure 15 illustrates the Web GUI for the hog farm management system which allows monitoring and controlling the the hog farm facility. In the GUI for the manager, the sensing values which are measured at the sensors installed inside and outside the hog farm appear in ⓑ, and ⓒ shows the control status of equipment at the hog farm and its conditions. ⓐ expresses the conditions of equipment in ⓒ as graphics. ⓓ is the part to control the CCTV stsme, ⓔ is the part to show the collected images through the CCTV, and ⓕ the part to enter standard values for automatic hog farm control.

It is possible to collect the hog farm environmental information and the hog farm video information through sensors and video monitoring cameras and constantly monitor/control the hog farm status through the user-intuitive GUI with these results.

**Figure 16.**
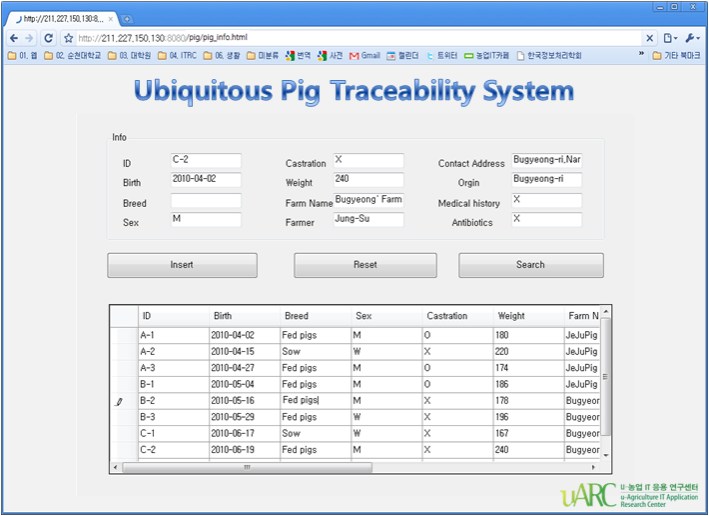
Ubiquitous Pig Traceability System GUI.

Figure 16 shows the Web GUI for monitoring pigs’ traceability data such as its identification, history and body information.

When the identification number is entered on the screen which displays pig’s history and body information, then the animal’s identification number, date of birth, place of birth, age, weight, class and disease history, *etc*., could be checked.

### 4.6. Implementation Result

**Figure 17.**
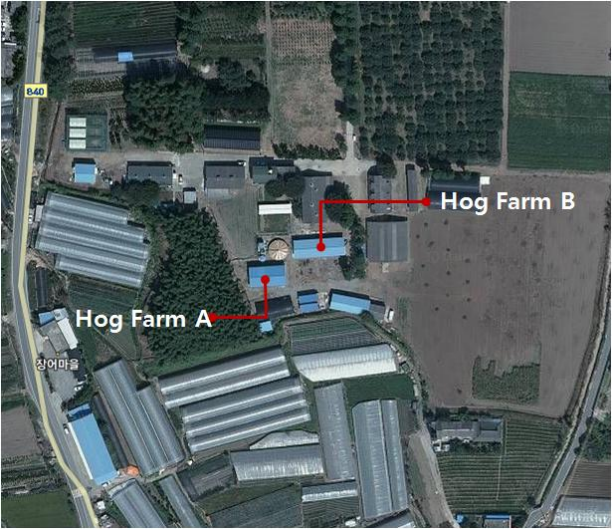
Location of hog Farm A and hog Farm B in Seo-myeon, Suncheon-si, Jeollanam-do, Korea.

Test beds were established at two hog farms located at the sites shown in Figure 17 in order to assess the proposed system. Wired sensors and the WSN were formed at hog farm A while wired sensors, WSN and the proposed ubiquitous hog farm system were installed at hog farm B. Both farms are located in the same area within 5 m distance.

**Figure 18.**
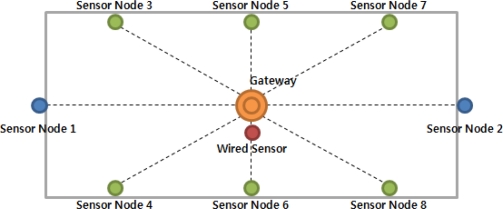
Wireless Sensor Network Topology.

Figure 18 shows the structure of sensors and gateways installed at the two hog farms. Sensor 1 and Sensor 2 are located outside of hog farm to measure the external temperature and humidity. Sensors 3 to 8 were installed inside the hog farm to measure the internal temperature and humidity.

Since WSN environmental sensors may provide wrong environment information about the hog farm, wired sensors were installed in both farms to compare the environmental information values obtained from the WSN, and WSN environmental sensors and wired sensors installed at each hog farm transmit their measured results to the server every 10 minutes.

**Figure 19.**
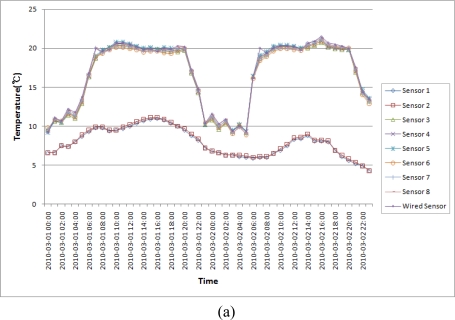

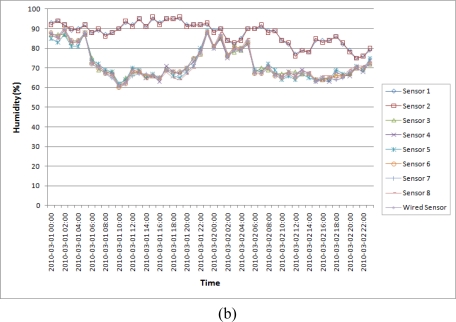
Temperature and Humidity Variation Graph of Hog Farm A. **(a)** Temperature Variation Graph of Hog Farm A (2010/03/02); **(b)** Humidity Variation Graph of Hog Farm A (2010/03/02).

Users shall maintain the breeding environment of the hog farm by controlling facility manually based on the measured data in the hog farm A whereas, in the hog farm B where the proposed ubiquitous hog farm system is installed, the CML shall control facility of the hog farm automatically in a way appropriate to each situation given after checking whether the control facility of the farm is operating or not by transmitting the control signal to the FML based on the inferred information. The CML shall create the inferred information by examining the situation and the surrounding environmental patterns of the farm comparing the collected information to the environment base values stored in the database via the DE and sends it to FML based on the information inferred from the EPL. Figure 19 is a temperature and humidity variation graph of the hog farm A.

As a result of measuring the data from hog farm A, we could find that user maintained the breeding environment of the hog farm by keeping a certain level of temperature and humidity because the user directly controlled the facility from 06:00 AM till 20:00 PM, but a sudden variation in temperature and humidity inside the hog farm occurred from 20:00 till 05:00 of the next day when user didn’t control the facility directly by himself. This sudden variation of temperature and humidity in the hog farm shall produce a big stress on the pigs, and if the level of the stress is serious, than it may lead the pig to die or a cause a disease like a cold to occur for the pigs which are weakend against disease [25,26].

**Figure 20.**
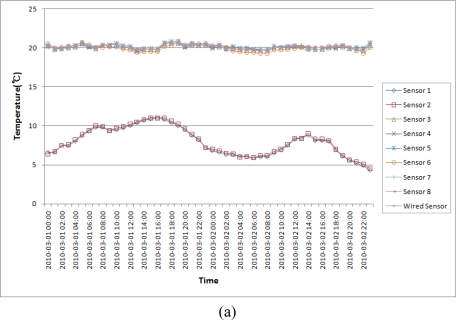

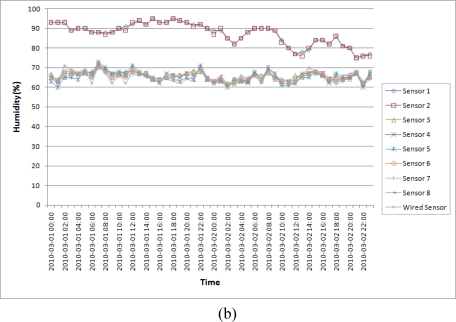
Temperature and Humidity Variation Graph of Hog Farm B. **(a)** Temperature Variation Graph of Hog Farm B (2010. 03. 02); **(b)** Humidity Variation Graph of Hog Farm B (2010. 03. 02).

Figure 20 is a temperature and humidity variation graph of hog farm B which is equipped and operated with the proposed ubiquitous hog farm system. In this farm B, the proposed system shall create a data forecast based on measured data, and compare the collected information to the environment base values stored in the database to infer the status of any relevant event and the patterns of its surrounding environment. Then it send the control signal to the facility management layer after checking whether the control facility of the hog farm is operating or not based on the inferred information from the EPL so that the control facility of the hog farm could be controlled appropriately for the situation given automatically.

As a result of measuring the hog farm B data by applying the proposed system, we could find that the proposed system always kept the breeding environment of the hog farm at a certain level of temperature and humidity because the hog farm facilities were automatically controlled based on the breeding environment values which were already set up. Therefore, we could find that the proposed system removed environmental factors such as extreme temperatures, humidity and various harmful gases in the hog farm in order to prevent diseases and maintain an optimal breeding environment.

In addition, we could find that the hog farm with the proposed system did maintain a more constant temperature and humidity than those of the hog farm without the proposed system, so consequently operating the proposed system would produce more efficient results.

Besides, since the implemented environment sensors of the hog farm may provide wrong information about the hog farm, we compared it to the environment information values obtained from wired sensors which were installed in the hog farm. As a result of measuring the hog farms, we could recognize that temperature and humidity data collected from WSN environmental sensors and that from wired sensors were nearly identical to each other and, we could see that the sensor nodes realized widely to apply the sensor network did work without any problems.

## 5. Conclusions

In this study, we proposed an ubiquitous hog farm system adopting wireless sensor network technology which to be applied to the pig industry in order to implement a systematic and scientific pig breeding technology.

The proposed system is composed of the physical layer to collect information about the hog farm and to control the environment, and the application layer which supports communication between the physical layer and the application layer, and lastly the middle layer which utilizes the information of the hog farm as a database and provides monitoring and control services to maintain the pig breeding environment in optimal status.

The system suggests that WSNs and CCTVs be installed in the hog farm to collect environmental and visual information which shall then help not only to monitor the hog farm via the Web from outside the farm, but also to manually control hog farm facilities in remote places. In addition, facilities shall be automatically controlled based on the breeding environment values which are already set up and SMS notice service shall provide users with additional convenience. Further, identification and individual information about the pigs shall be collected and managed by use of RFID tags through which a efficient traceability management of the pigs is available.

This proposed system provides environmental monitoring and traceability management for users and it can also be applied for warehouse management, greenhouse management, cattle shed management, *etc.*

In order to demonstrate the system, we operated test beds at two hog farms, through which we could recognize that the hog farm that adopted the proposed system provided a user with a better breeding environment and convenience by maintaining a constant temperature and humidity status at the hog farm. However, the environmental sensors of the proposed system suffered from corrosion caused by ammonia and the humidity of the hog farm. In addition, the sensing period of environmental sensors were set too short for real-time monitoring of the hog farm environment, so the proposed system suffered from a life problem with the sensors energy. When these problems occur, the environmental sensors cannot collect environmental information about the hog farm and the proposed system cannot provide users with the desired information.

A future study is scheduled to improve the reliability of data transmission in the wireless sensor network and the energy efficiency of sensor nodes. We will also apply the proposed system in various agricultural environments and extend the functionality of the system.

## Figures and Tables

**Table 1.** Hardware type and specifications of WSN Environmental Sensor Nodes.

**Hardware Type**	**Hardware Specifications**

Processor (MSP430F1611)	Data Bus Width: 16 bit
	Program Memory Size: 48 KB
	Data RAM Size: 10 KB
	Maximum Clock Frequency: 8 MHz
	On-Chip ADC: 8-chx12-bit
	On-Chip DAC: 2-chx12-bit
	Number of Programmable I/Os: 48
	Interface Type: USART
	Operating Supply Voltage: 1.8 V to 3.6 V
	Maximum Operating Temperature: 85 °C
	Minimum Operating Temperature: −40 °C

RF Device (CC2420)	Radio Frequency (Mhz): 2,400
	Max. Data Rate (kbits/sec): 250
	Antenna: PCB Antenna or SMA

Temperature Humidity Sensor (SHT-71)	Vmax (VDD): 2.4–5.5
	Humidity range: 0–100%
	RH Humidity Accuracy: ±3% RH (20–80% RH)
	Repeatability: ±0.1% RH
	Temperature Accuracy: ±0.4 °C @ 25 °C

Luminance Sensor (GL5547)	Vmax (VDC): 150
	Pmax (mW): 100
	Ambient Temp (°C): −30∼+70
	Spectral Peak (nm): 540
	Response Time (ms): Rise 20, Decay 30

**Table 2.** Hardware type and specifications of Wired Integrated Sensor Nodes.

**Hardware Type**	**Hardware Specifications**

CO_2_ Sensor	Measurement methods: NDIR
	Measuring range: 0 ∼ 3,000 ppm
	Precision: ±3%
	Response time: 0 ∼ 80% < 2 min
	Update interval: 2 Seconds
	Warm-up time: @25 °C < 3 min
	Operating Temperature: 0 ∼ 50 °C
	Operating Humidity: 0 ∼ 95% RH

Temperature Sensor	Measuring range: −25 ∼ 65°C
	Resolution: 0.01 °C
	Repeat accuracy: ±0.1 °C
	Response time: (Min) 5 ∼ (Max) 30 Seconds

Humidity Sensor	Measuring range: 0∼100% RH
	Resolution: 0.03% RH
	Repeat accuracy: ±0.1%RH
	Response time: 4 Seconds

**Table 3.** Hardware type and specifications of the RFID.

**Item**	**Value**
Frequency:	2,410–2,472 MHz ISM band—FCC certified for unlicensed use
Antennas:	Separate transmit and receive ports, 50 Ohm, reverse TNC connector
Power supply:	12 VDC, 2 A (unregulated)
Power consumption:	25 Watts
Communication interface:	RS232; 9-pin, Sub D (female)
Communication settings:	Baud 115200, Data bits 8, Stop Bits 1, Parity None
LAN interface:	10baseT Ethernet
Inputs/outputs:	4 programmable logic I/O
Indicators:	Power, RF, Sniff, Lock
Operating Temperature:	−20 °C to 55 °C
Dimensions (H x W x D):	7 × 10 × 2 inches
Weight:	2.06 Kg (4.6 lbs)

**Table 4.** Web GUI Development Environment.

	**Type & Version**
H/W	Samsung Xeon 3.2 Ghz × 1 GB
OS	Fedora Linux 2.6.27
DB	Mysql 5.0
WAS	Tomcat 6.0.20
